# Unusual Complication of Suprapubic Cystostomy in a Male Patient with Tetraplegia: Traction on Foley Catheter Leading to Extrusion of Foley Balloon from Urinary Bladder and Suprapubic Urinary Fistula – Importance of Securely Anchoring Suprapubic Catheter with Adhesive Tape or BioDerm Tube Holder

**DOI:** 10.1100/tsw.2007.253

**Published:** 2007-09-17

**Authors:** Subramanian Vaidyanathan, Peter L. Hughes, Bakul M. Soni

**Affiliations:** ^1^ Regional Spinal Injuries Centre, District General Hospital, Southport, Merseyside PR8 6PN, UK; ^2^ Department of Radiology, District General Hospital, Southport, Merseyside PR8 6PN, UK

**Keywords:** suprapubic cystostomy, urinary bladder, spinal cord injury

## Abstract

Suprapubic cystostomy is recommended to patients with neuropathic bladder to prevent complications of long-term urethral catheter drainage. We present a 50-year-old male patient with tetraplegia who had long-term urethral catheter drainage. Following flexible cystoscopy, he developed a urine leak from the right side of the scrotum. Suprapubic cystostomy was performed. After suprapubic cystostomy, the urinary fistula healed completely. A follow-up cystourethrogram confirmed an intact urethra with no leak of contrast. Six weeks later, this patient presented with a hole below the suprapubic cystostomy through which a small amount of urine was leaking. A keyhole dressing had been applied around the suprapubic catheter and the catheter was hanging loosely, thus permitting traction on the catheter, especially when the urine bag was full. Computerised tomography of the pelvis showed extrusion of the Foley balloon from the urinary bladder, but the tip of the catheter was still located within the bladder. The extruded catheter was removed and a Foley catheter was inserted, ensuring that the balloon was inflated within the urinary bladder. The suprapubic catheter was secured firmly to the anterior abdominal wall with a BioDerm Tube Holder, thus preventing any traction on the catheter or Foley balloon. The urine leak through the hole below the suprapubic cystostomy stopped and the sinus healed. This case illustrates the need to anchor the suprapubic catheter securely to the anterior abdominal wall with adhesive tape or BioDerm Tube Holder to prevent traction and consequent displacement of the catheter or Foley balloon.

## BACKGROUND

Long-term, indwelling, urethral catheters may cause local adverse effects, such as erosion of the penile urethra, leading to urethral split (catheter-induced hypospadias). In female patients, who manage the neuropathic bladder by indwelling urethral catheter drainage, the balloon of the Foley catheter may erode the bladder neck. The risk of mechanical damage to the urethra and bladder neck by an indwelling urethral catheter increases when the catheter is not held securely, thus allowing traction to be applied inadvertently to the catheter and Foley balloon. Suprapubic cystostomy is recommended to prevent complications of long-term urethral catheter drainage[1].

We present a male patient with tetraplegia, who developed an unusual complication of a suprapubic cystostomy manifesting as a urinary fistula below the site of the suprapubic cystostomy.

## CASE REPORT

A 50-year-old male patient with tetraplegia was managing a neuropathic bladder by a long-term, indwelling, urethral catheter. He developed urethral discharge and a flexible cystoscopy was performed in another hospital. Following cystoscopy, there was swelling of the right side of the scrotum. Two days later, the swelling burst open, leaking watery fluid. Retrograde urethrography showed irregularity in the outline of the penile urethra and a contrast leak, leading to a large irregular cavity to the right side in the scrotum. Suprapubic cystostomy was performed following which the urinary fistula healed. Follow-up cystourethrography showed no extravasation of contrast from the urethra.

Four months after undergoing suprapubic cystostomy, the patient presented with a hole of 1 cm in diameter just below the suprapubic cystostomy (Fig. 1). A small amount of urine was leaking through this hole. The suprapubic catheter was draining less than the usual amount of urine. A keyhole dressing had been applied to the site of the suprapubic cystostomy, allowing the catheter to hang loosely. In the absence of any dressing or device to hold the suprapubic catheter, the urine bag was pulling the catheter. When the urine bag was full, the weight of the bag exerted considerable traction on the suprapubic catheter.

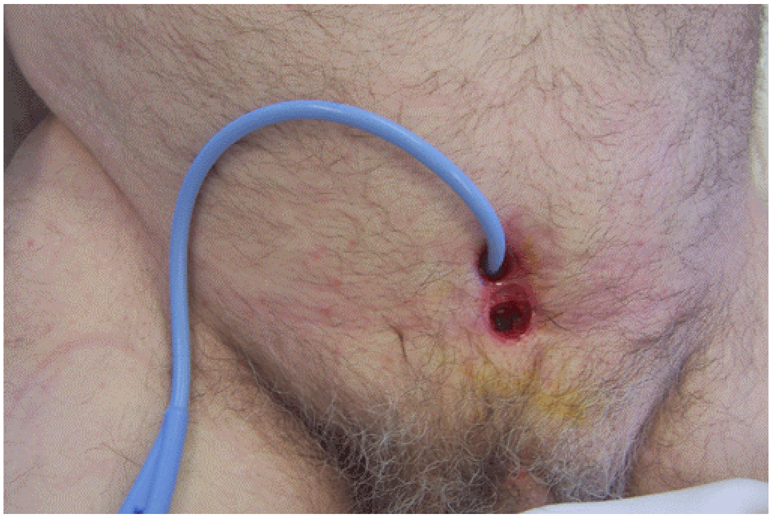

**FIGURE 1.** Clinical photograph of the patient. The suprapubic catheter was hanging loosely. There was a hole below the suprapubic cystostomy through which small amount of urine was leaking.

Computerised tomography (CT) of the pelvis was performed to assess the anatomical relationship between the urinary bladder, the hole below the suprapubic cystostomy, and the suprapubic catheter. CT showed the balloon of the suprapubic catheter to lie outside the urinary bladder (Fig. 2). The hole, situated below the suprapubic catheter, tracked inferiorly for a few centimetres to end in a 2.5-cm diameter inflammatory area anterior to the upper border of the symphysis pubis.

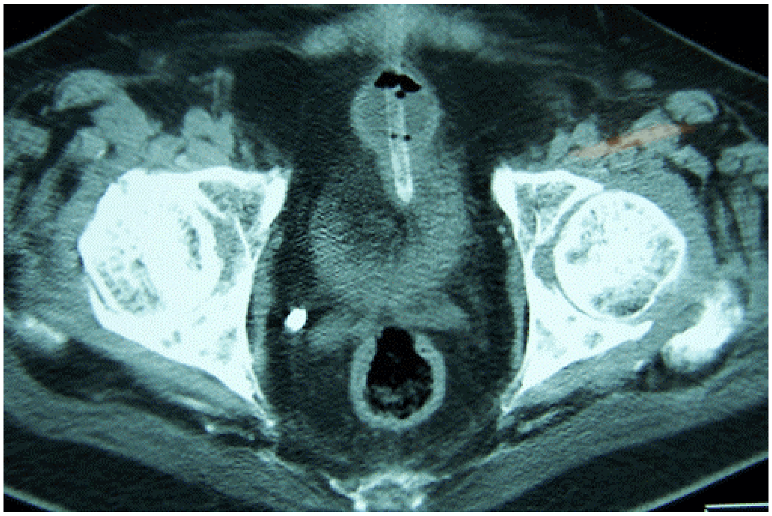

**FIGURE 2**. Computerised tomography of the pelvis. The balloon of the Foley catheter was lying outside the urinary bladder. The tip of the Foley catheter was located within the lumen of the urinary bladder. This would explain why the suprapubic catheter was still draining urine, albeit less than usual amounts.

The extruded catheter was removed and a new catheter was inserted, ensuring that the balloon was inflated within the urinary bladder. The suprapubic catheter was fixed firmly to the abdominal wall with a BioDerm Tube Holder (Fig. 3). The urine leak from the suprapubic fistula stopped and the sinus healed.

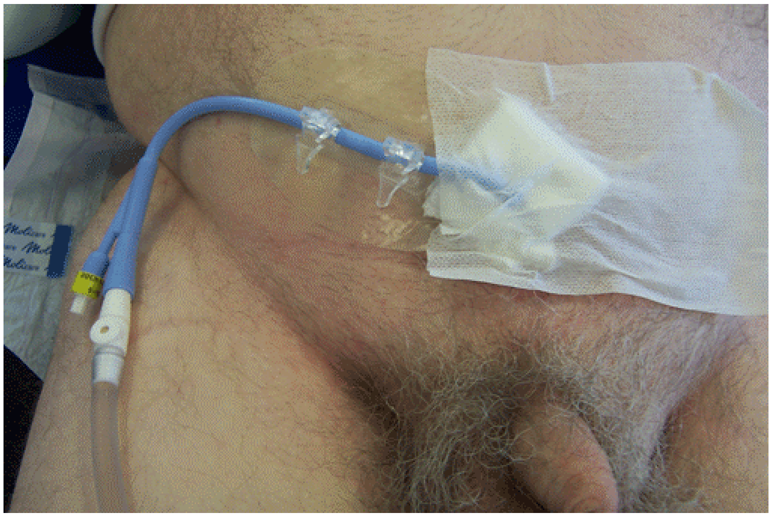

**FIGURE 3**. Clinical photograph of the patient after applying BioDerm Tube Holder. A dressing and BioDerm Tube Holder hold the suprapubic catheter securely, thus preventing any traction on the Foley catheter.

## COMMENT

Traction on the Foley catheter resulted in extrusion of the Foley balloon from the urinary bladder and, consequently, leakage of urine to the area anterior to the upper border of the symphysis pubis. The extruded balloon of the Foley catheter was fitting snugly to the cystostomy hole and did not allow exit of urine around the suprapubic catheter. Therefore, urine found its way to the exterior through a separate hole below the suprapubic cystostomy (Fig. 1). When the extruded catheter was removed and a new catheter was inserted with the balloon inflated within the urinary bladder, the urine leak ceased.

This case illustrates the need to apply dressing or a device to anchor a suprapubic catheter to the abdominal wall in order to prevent traction on the catheter. We used a BioDerm Tube Holder in this patient to anchor the suprapubic catheter to the abdominal wall. The BioDerm Tube Holder utilizes a proprietary hydrocolloid adhesive to adhere to the skin. The design of this tube holder allows for repeated removal and replacement of tubing without trauma to the patient or the tube. The BioDerm Tube Holder is not yet available on prescription in the National Health Service in the U.K. Since patients are unable to get the BioDerm Tube Holder in the community, the Regional Spinal Injuries Centre, Southport, procures this device and supplies it to the Centre’s patients who have a suprapubic cystostomy. Each tube holder costs £9.28 (inclusive of value added tax). Usually, spinal cord injury patients change the BioDerm Tube Holder once a week. We found the BioDerm Tube Holders reliable and cost effective, as they prevent complications that result from accidental catheter displacement. A simple adhesive tape can be used to anchor the catheter, but tapes, particularly those containing rubber, can cause irritant and allergic reactions in susceptible patients. Tapes with synthetic adhesives overcome this problem, but they too may sometimes be associated with reactions. Adhesive tapes that are occlusive may cause skin maceration[2]. Spinal cord injury patients, who have neuropathic skin, are vulnerable for developing adverse reaction to adhesive tapes. We have been using permeable, apertured, nonwoven, synthetic adhesive tape for dressing over suprapubic catheters and to anchor the catheters in selected patients. This particular patient was not advised to secure the suprapubic catheter with adhesive tape or a tube holder, which proved to be a costly mistake.

## CONCLUSION

A simple error of omission on the part of health professionals resulted in a serious complication in a tetraplegic patient. His suprapubic catheter was not held securely, thus allowing traction on the catheter, which led to extrusion of the Foley balloon from the urinary bladder. There was a urine leak from the bladder, resulting in a suprapubic urinary fistula. We learn an important lesson from this case, i.e., that we should advise *all* patients with a suprapubic cystostomy to anchor the suprapubic catheters to the abdominal wall with adhesive tape or a BioDerm Tube Holder in order to prevent traction and accidental displacement of the suprapubic catheter.
